# Vaccination Coverage by Age 24 Months Among Children Born in 2017 and 2018 — National Immunization Survey-Child, United States, 2018–2020

**DOI:** 10.15585/mmwr.mm7041a1

**Published:** 2021-10-15

**Authors:** Holly A. Hill, David Yankey, Laurie D. Elam-Evans, James A. Singleton, Natalie Sterrett

**Affiliations:** 1 Immunization Services Division, National Center for Immunization and Respiratory Diseases, CDC.

Immunization is a safe and cost-effective means of preventing illness in young children and interrupting disease transmission within the community.* The Advisory Committee on Immunization Practices (ACIP) recommends vaccination of children against 14 diseases during the first 24 months of life (*1*). CDC uses National Immunization Survey-Child (NIS-Child) data to monitor routine coverage with ACIP-recommended vaccines in the United States at the national, regional, state, territorial, and selected local levels.^†^ CDC assessed vaccination coverage by age 24 months among children born in 2017 and 2018, with comparisons to children born in 2015 and 2016. Nationally, coverage was highest for ≥3 doses of poliovirus vaccine (92.7%); ≥3 doses of hepatitis B vaccine (HepB) (91.9%); ≥1 dose of measles, mumps, and rubella vaccine (MMR) (91.6%); and ≥1 dose of varicella vaccine (VAR) (90.9%). Coverage was lowest for ≥2 doses of influenza vaccine (60.6%). Coverage among children born in 2017–2018 was 2.1–4.5 percentage points higher than it was among those born in 2015–2016 for rotavirus vaccine, ≥1 dose of hepatitis A vaccine (HepA), the HepB birth dose, and ≥2 doses of influenza vaccine. Only 1.0% of children had received no vaccinations by age 24 months. Disparities in coverage were seen for race/ethnicity, poverty status, and health insurance status. Coverage with most vaccines was lower among children who were not privately insured. The largest disparities between insurance categories were among uninsured children, especially for ≥2 doses of influenza vaccine, the combined 7-vaccine series,^ §^ and rotavirus vaccination. Reported estimates reflect vaccination opportunities that mostly occurred before disruptions resulting from the COVID-19 pandemic. Extra efforts are needed to ensure that children who missed vaccinations, including those attributable to the COVID-19 pandemic, receive them as soon as possible to maintain protection against vaccine-preventable illnesses.

CDC conducts the NIS-Child annually as a random-digit–dialed mobile telephone survey^¶^ of parents or guardians of children aged 19–35 months. Interviewers collect sociodemographic information and then request consent to contact the child’s vaccination providers. When consent is obtained, a survey is mailed to each provider requesting the child’s vaccination information. A synthesized, comprehensive vaccination history is created to estimate vaccination coverage. Children born in 2017 and 2018 were identified from data collected during 2018–2020, resulting in a sample of 29,114 children with adequate provider data.** For data collected in 2020, the household response rate^††^ was 22.5%, and adequate provider data were obtained for 54.2% of households with completed interviews. Kaplan-Meier (time to event) analysis was used to estimate coverage by age 24 months for most vaccines. Exceptions include the HepB birth dose, measured as the proportion of children who received a dose of HepB by age 3 days, and rotavirus vaccine, assessed at age 8 months to reflect the maximum ACIP-recommended administration age. Coverage with ≥2 HepA doses was estimated by age 35 months (the maximum age included in the survey) because the recommended immunization schedule permits administration of the second dose as late as age 41 months. Coverage estimates for children born in 2017 and 2018 were compared with estimates for children born in 2015 and 2016. All coverage differences were assessed on weighted data using t-tests for comparing two independent proportions; p-values <0.05 were considered statistically significant. Analyses were performed using SAS (version 9.4; SAS Institute) and SUDAAN (version 11; RTI International). This activity was reviewed by CDC and was conducted consistent with applicable federal law and CDC policy.^§§^

## National Vaccination Coverage

Among children born in 2017 and 2018, coverage by age 24 months exceeded 90% for ≥3 doses of poliovirus vaccine (92.7%), ≥3 doses of HepB (91.9%), ≥1 dose of MMR (91.6%), and ≥1 dose of VAR (90.9) (Table 1). The lowest coverage was observed for ≥2 doses of influenza vaccine (60.6%), although influenza vaccination coverage increased 4.5 percentage points compared with coverage among children born in 2015 and 2016. Coverage increased 4.0 percentage points for the HepB birth dose and 2.1 percentage points for both rotavirus vaccine and ≥1 dose of HepA. The percentage of children who received no vaccinations by age 24 months decreased from 1.4% among those born in 2015 and 2016 to 1.0% among those born in 2017 and 2018.

**TABLE 1:** Estimated vaccination coverage by age 24 months,* among children born during 2015–2018, by selected vaccines and doses — National Immunization Survey-Child, United States, 2016–2020

Vaccine/Dose	% (95% CI)		
	Birth years^†^		Difference (2015–2016 to 2017–2018)
	2015–2016	2017–2018	
**DTaP^§^**			
≥3 doses	93.8 (93.2 to 94.3)	93.7 (93.1 to 94.3)	−0.1 (−0.9 to 0.7)
≥4 doses	80.5 (79.5 to 81.5)	81.6 (80.6 to 82.5)	1.1 (−0.3 to 2.4)
**Poliovirus (≥3 doses)**	92.5 (91.9 to 93.1)	92.7 (92.1 to 93.3)	0.2 (−0.7 to 1.0)
**MMR (≥1 dose)^¶^**	90.8 (90.1 to 91.4)	91.6 (90.8 to 92.2)	0.8 (−0.2 to 1.7)
Hib**			
Primary series	92.7 (92.1 to 93.4)	92.9 (92.3 to 93.5)	0.2 (−0.7 to 1.1)
Full series	79.8 (78.8 to 80.8)	80.2 (79.2 to 81.2)	0.4 (−1.0 to 1.8)
**HepB**			
Birth dose^††^	74.4 (73.3 to 75.6)	78.4 (77.4 to 79.4)	4.0 (2.4 to 5.5)^§§^
≥3 doses	91.0 (90.2 to 91.7)	91.9 (91.2 to 92.5)	0.9 (−0.1 to 1.9)
**VAR (≥1 dose)^¶^**	90.3 (89.6 to 90.9)	90.9 (90.2 to 91.6)	0.6 (−0.4 to 1.6)
**PCV**			
≥3 doses	91.9 (91.2 to 92.6)	92.4 (91.8 to 93.1)	0.5 (−0.4 to 1.4)
≥4 doses	81.2 (80.2 to 82.2)	82.3 (81.4 to 83.2)	1.1 (−0.3 to 2.4)
**HepA**			
≥1 dose	84.9 (84.1 to 85.8)	87.0 (86.2 to 87.9)	2.1 (0.9 to 3.3)^§§^
≥2 doses (by age 35 mos)	76.3 (74.9 to 77.7)	77.7 (76.1 to 79.1)	1.4 (−0.7 to 3.4)
**Rotavirus (by age 8 mos)^¶¶^**	73.6 (72.4 to 74.7)	75.6 (74.6 to 76.7)	2.1 (0.5 to 3.6)^§§^
Influenza (≥2 doses)***	56.0 (54.8 to 57.2)	60.6 (59.4 to 61.8)	4.5 (2.9 to 6.2)^§§^
**Combined 7-vaccine series^†††^**	69.0 (67.8 to 70.1)	70.5 (69.4 to 71.7)	1.6 (0.0 to 3.2)
**No vaccinations**	1.4 (1.2 to 1.6)	1.0 (0.8 to 1.1)	−0.4 (−0.7 to −0.2)^§§^

**Abbreviations:** CI = confidence interval; DTaP = diphtheria, tetanus toxoids, and acellular pertussis vaccine; HepA = hepatitis A vaccine; HepB = hepatitis B vaccine; Hib = *Haemophilus influenzae* type b conjugate vaccine; MMR = measles, mumps, and rubella vaccine; PCV = pneumococcal conjugate vaccine; VAR = varicella vaccine.

* Includes vaccinations received by age 24 months (before the day the child turns 24 months), except for the HepB birth dose, rotavirus vaccination, and ≥2 HepA doses by age 35 months. For all vaccines except the HepB birth dose and rotavirus vaccination, the Kaplan-Meier method was used to estimate vaccination coverage to account for children whose vaccination history was ascertained before age 24 months (35 months for ≥2 HepA doses).

^†^ Data for the 2015 birth year are from survey years 2016, 2017, and 2018; data for the 2016 birth year are from survey years 2017, 2018, and 2019; data for the 2017 birth year are from survey years 2018, 2019, and 2020; data for birth year 2018 are considered preliminary and come from survey years 2019 and 2020 (data from survey year 2021 are not yet available).

^§^ Includes children who might have received diphtheria and tetanus toxoids vaccine or diphtheria, tetanus toxoids, and pertussis vaccine.

^¶^ Includes children who might have received measles, mumps, rubella, and varicella combination vaccine.

** Hib primary series: receipt of ≥2 or ≥3 doses, depending on product type received; full series: primary series and booster dose, which includes receipt of ≥3 or ≥4 doses, depending on product type received.

^††^ One dose HepB administered from birth through age 3 days.

^§§^ Statistically significantly different from zero at p<0.05.

^¶¶^ Includes ≥2 doses of Rotarix monovalent rotavirus vaccine, or ≥3 doses of RotaTeq pentavalent rotavirus vaccine. (If any dose in the series is either RotaTeq or unknown, default to 3-dose series.) The maximum age for the final rotavirus dose is 8 months, 0 days.

*** Doses must be ≥24 days apart (4 weeks with a 4-day grace period); doses could have been received during two influenza seasons.

^†††^ The combined 7-vaccine series (4:3:1:3*:3:1:4) includes ≥4 doses of DTaP, ≥3 doses of poliovirus vaccine, ≥1 dose of measles-containing vaccine, the full series of Hib (≥3 or ≥4 doses, depending on product type), ≥3 doses of HepB, ≥1 dose of VAR, and ≥4 doses of PCV.

## Vaccination by Selected Sociodemographic Characteristics and Geographic Locations

Coverage by age 24 months was lower for most vaccines among children who did not have private health insurance (Table 2). The largest coverage disparities were observed for receipt of ≥2 doses of influenza vaccine, rotavirus vaccination, and the combined 7-vaccine series. The largest disparities between insurance categories were for uninsured children; percentage point differences between uninsured and privately insured children ranged from 9.2 (≥3 HepB doses) to 37.8 (≥2 influenza vaccine doses) and were present for all vaccines except the HepB birth dose. The percentage of children who received no vaccinations by age 24 months was higher among uninsured (3.3%) than privately insured (0.8%) children. Coverage was lower for both Black and Hispanic children compared with White children for most vaccines (Supplementary Table 1, https://stacks.cdc.gov/view/cdc/110375). Coverage was lower among children living below the poverty level than among those living at or above the poverty level for all vaccines except the HepB birth dose; fewer disparities were found by Metropolitan Statistical Area (MSA)^¶¶^ (Supplementary Table 2, https://stacks.cdc.gov/view/cdc/110376). Wide variation in estimated vaccination coverage was observed by jurisdiction (Supplementary Table 3, https://stacks.cdc.gov/view/cdc/110377), especially for ≥2 doses of influenza vaccine, with estimates ranging from 37.7% (Alabama) to 80.2% (Massachusetts) (Figure).

**TABLE 2:** Estimated vaccination coverage by age 24 months* among children born during 2017–2018,^†^ by selected vaccines and doses and health insurance status^§^ — National Immunization Survey-Child, United States, 2018–2020

Vaccine/Dose	Health insurance status, % (95% CI)			
	Private only (referent) (n = 15,686)	Any Medicaid (n = 10,331)	Other insurance (n = 2,280)	Uninsured (n = 817)
**DTaP^¶^**				
≥3 doses	96.3 (95.7–96.9)	92.1 (91.0–93.0)**	92.3 (89.9–94.2)**	85.1 (80.9–88.7)**
≥4 doses	87.7 (86.5–88.8)	77.7 (76.1–79.3)**	78.2 (74.7–81.5)**	61.9 (55.2–68.7)**
**Poliovirus (≥3 doses)**	95.4 (94.6–96.0)	91.0 (89.9–92.1)**	91.2 (88.9–93.3)**	83.9 (79.7–87.8)**
**MMR (≥1 dose)^††^**	94.4 (93.5–95.1)	89.8 (88.6–90.9)**	90.3 (87.3–92.8)**	82.3 (76.8–87.2)**
**Hib^§§^**				
Primary series	95.8 (95.1–96.4)	91.2 (90.1–92.3)**	91.4 (89.0–93.5)**	82.4 (77.3–86.9)**
Full series	86.8 (85.6–87.9)	75.8 (74.1–77.5)**	77.4 (73.8–80.9)**	61.5 (54.9–68.1)**
**HepB**				
Birth dose^¶¶^	79.4 (78.0–80.7)	78.1 (76.4–79.7)	75.0 (71.3–78.5)**	76.5 (70.6–81.5)
≥3 doses	93.6 (92.7–94.4)	91.0 (89.9–92.1)**	90.5 (88.3–92.5)**	84.4 (80.0–88.3)**
**VAR (≥1 dose)^††^**	93.3 (92.4–94.2)	89.6 (88.4–90.8)**	89.8 (87.4–92.0)**	78.2 (72.2–83.6)**
**PCV**				
≥3 doses	95.3 (94.5–95.9)	90.7 (89.6–91.7)**	90.9 (88.5–93.0)**	83.1 (78.7–87.0)**
≥4 doses	89.2 (88.1–90.2)	77.7 (76.1–79.3)**	79.3 (75.7–82.6)**	62.2 (55.7–68.7)**
**HepA**				
≥1 dose	89.2 (88.1–90.3)	85.9 (84.6–87.2)**	87.5 (84.9–89.8)	72.8 (66.8–78.6)**
≥2 doses (by age 35 mos)	82.4 (80.4–84.2)	74.9 (72.4–77.2)**	78.4 (72.7–83.6)	—***
**Rotavirus (by age 8 mos)^†††^**	84.7 (83.4–85.8)	68.8 (67.0–70.6)**	73.9 (70.3–77.2)**	55.7 (49.0–62.1)**
**Influenza (≥2 doses)^§§§^**	74.2 (72.8–75.6)	49.9 (47.9–51.8)**	57.8 (53.6–62.0)**	36.4 (30.5–43.0)**
**Combined 7-vaccine series^¶¶¶^**	78.3 (76.8–79.6)	65.6 (63.7–67.4)**	65.7 (61.7–69.7)**	48.3 (41.8–55.2)**
**No vaccinations**	0.8 (0.6–1.0)	1.0 (0.8–1.3)	0.9 (0.5–1.4)	3.3 (1.9–5.4)**

**Abbreviations:** CI = confidence interval; DTaP = diphtheria, tetanus toxoids, and acellular pertussis vaccine; HepA = hepatitis A vaccine; HepB = hepatitis B vaccine; Hib = *Haemophilus influenzae* type b conjugate vaccine; MMR = measles, mumps, and rubella vaccine; PCV = pneumococcal conjugate vaccine; VAR = varicella vaccine.

* Includes vaccinations received by age 24 months (before the day the child turns 24 months), except for the HepB birth dose, rotavirus vaccination, and ≥2 HepA doses by age 35 months. For all vaccines except the HepB birth dose and rotavirus vaccination, the Kaplan-Meier method was used to estimate vaccination coverage to account for children whose vaccination history was ascertained before age 24 months (35 months for ≥2 HepA doses).

^†^ Data for the 2017 birth year are from survey years 2018, 2019, and 2020; data for the 2018 birth year are considered preliminary and come from survey years 2019 and 2020 (data from survey year 2021 are not yet available).

^§^ Children’s health insurance status was reported by parent or guardian. “Other insurance” includes the Children’s Health Insurance Program (CHIP), military insurance, coverage via the Indian Health Service, and any other type of health insurance not mentioned elsewhere.

^¶^ Includes children who might have received diphtheria and tetanus toxoids vaccine or diphtheria, tetanus toxoids, and pertussis vaccine.

** Statistically significant (p<0.05) difference compared with the referent group.

^††^ Includes children who might have received measles, mumps, rubella, and varicella combination vaccine.

^§§^ Hib primary series: receipt of ≥2 or ≥3 doses, depending on product type received; full series: primary series and booster dose, which includes receipt of ≥3 or ≥4 doses, depending on product type received.

^¶¶^ One dose HepB administered from birth through age 3 days.

*** Estimate not available because the unweighted sample size for the denominator was <30, or 95% CI half width/estimate >0.588, or 95% CI half width was ≥10.

^†††^ Includes ≥2 doses of Rotarix monovalent rotavirus vaccine or ≥3 doses of RotaTeq pentavalent rotavirus vaccine. (If any dose in the series is either RotaTeq or unknown, default to 3-dose series.) The maximum age for the final rotavirus dose is 8 months, 0 days.

^§§§^ Doses must be at least 24 days apart (4 weeks with a 4-day grace period); doses could have been received during two influenza seasons.

^¶¶¶^ The combined 7-vaccine series (4:3:1:3*:3:1:4) includes ≥4 doses of DTaP, ≥3 doses of poliovirus vaccine, ≥1 dose of measles-containing vaccine, the full series of Hib (≥3 or ≥4 doses, depending on product type), ≥3 doses of HepB, ≥1 dose of VAR, and ≥4 doses of PCV.

**FIGURE:**
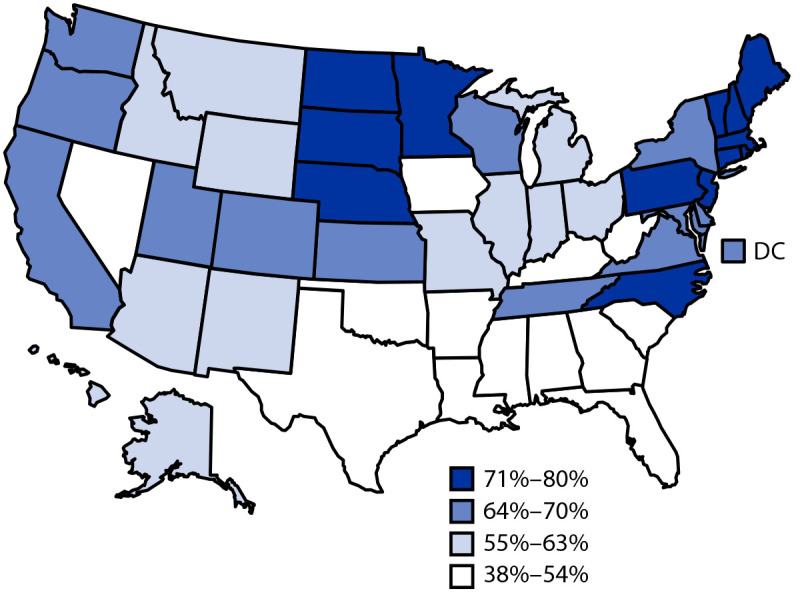
Estimated vaccination coverage with ≥2 doses of influenza vaccine* by age 24 months, among children born during 2017–2018^†^ — National Immunization Survey-Child, United States, 2018–2020 **Abbreviation:** DC = District of Columbia. * Doses must be ≥24 days apart (4 weeks with a 4-day grace period); doses could have been received during two influenza seasons. ^†^ Data for the 2017 birth year are from survey years 2018, 2019, and 2020; data for the 2018 birth year are considered preliminary and come from survey years 2019 and 2020 (data from survey year 2021 are not yet available).

## Discussion

Among children born during 2017–2018, national coverage with most routine childhood vaccines remained stable,*** with some increases compared with 2015–2016. Although recent data show a decrease in the percentage of children receiving no vaccinations by age 24 months, no evidence has been observed of a trend across birth cohorts from 2011 to 2018.^†††^ Coverage estimates varied substantially by sociodemographic characteristics. Children with private insurance had higher coverage than did all other insured children. Coverage among children who did not have private health insurance was 9.2 to 37.8 percentage points lower than that for children with private insurance (except for the HepB birth dose) and a higher likelihood of being completely unvaccinated compared with children with private insurance. Other characteristics associated with lower vaccination coverage include living below the federal poverty level and being of Black race or Hispanic ethnicity. Observed disparities by MSA were less frequent and not consistent in direction, as was the case with other sociodemographic variables. Relationships among coverage disparities and overall coverage are complex: national coverage increased for four vaccines and did not decrease for any; however, the number of vaccines with statistically significant disparities increased (*2*).

The presence of widespread and often substantial disparities in coverage with routinely recommended vaccines indicates a need for improvement to achieve equity in the national childhood vaccination program (*3*). Important barriers to overcome include access to vaccination services, financial challenges, missed vaccination opportunities, and vaccine hesitancy. Some parents might find it difficult to identify a provider, arrange transportation, and take time off from work to attend a vaccination visit. The lower coverage observed in children living below the federal poverty level and those without private health insurance might be attributable in part to these factors as well as financial challenges. The Vaccines for Children (VFC) program^§§§^ covers the cost of all recommended vaccines for eligible children; however, parents might not be aware of the program or how to access it. Children should receive all vaccinations for which they are eligible at each provider visit; elimination of missed vaccination opportunities has been shown to increase potentially achievable coverage across sociodemographic characteristics including poverty level and health insurance status (*4*). Vaccine hesitancy has been shown to be more common among low-income families and among parents of non-Hispanic Black (versus non-Hispanic White) children (*5*). CDC has developed the Vaccinate with Confidence strategy (*6*), which identifies activities designed to strengthen vaccine confidence and prevent outbreaks of vaccine-preventable diseases in the United States.

The findings in this report are subject to at least three limitations. First, the household interview response rate was low (22.5%), and adequate provider data were available for only 54.2% of those with completed interviews. This could introduce selection bias if study respondents and nonrespondents differed on factors related to vaccination coverage. Second, although the weighting is designed to adjust for nonresponse and for households without mobile phones, this adjustment might not completely eliminate bias. Finally, coverage might be underestimated because of incomplete provider-reported vaccination histories. Total survey error (*7*) was assessed using 2019 survey data,^¶¶¶^ concluding that coverage with ≥4 doses of DTaP and ≥1 dose of MMR were each underestimated by two to three percentage points and coverage with the combined 7-vaccine series was underestimated by nine percentage points. However, a meaningful change in total survey error from 2019 to 2020 was considered unlikely.****

Concern has been raised about the negative impact of the COVID-19 pandemic on routine childhood vaccination in the United States, beginning in March 2020 when the pandemic was declared a national emergency (*8*,*9*). The findings in this report primarily reflect opportunity for vaccination that occurred before disruption related to COVID-19 because most children born before 2019 were aged ≥19 months by March 2020. From other data sources, decreases in both vaccine ordering and administration have been documented, including substantial declines in doses of DTaP and MMR administered to children aged 0–23 months during March–September 2020 compared with the same period in 2019 (*9*). Stay-at-home orders were common during this time, and parents might have avoided seeking routine care for their children because of a fear of contracting COVID-19 at health care facilities or in the community. Some rebound in vaccine administration to young children has been observed following multiple communications to health care providers emphasizing the importance of continued routine vaccination (*10*). This might be particularly important for influenza vaccine, for which coverage has been lower compared with other vaccines recommended for children. SARS-CoV-2, the virus that causes COVID-19, and influenza are likely to be co-circulating this fall and winter, which could put considerable strain on the public health and medical systems in the United States. Persistent disparities in vaccination coverage by health insurance status, race and ethnicity, and poverty status indicate that improvement is needed to achieve equity in the national childhood vaccination program. Efforts by health care providers and parents are needed to ensure that all children are protected from vaccine-preventable diseases.

**Summary**

**What is already known about this topic?**

The National Immunization Survey-Child monitors coverage with vaccines recommended by the Advisory Committee on Immunization Practices for children during the first 24 months of life to prevent 14 diseases.

**What is added by this report?**

Coverage with most childhood vaccines among children born in 2017 and 2018 was lower among those who were uninsured, Black, Hispanic, or living below the federal poverty level than it was among those who were privately insured, White, or living at or above the poverty level.

**What are the implications for public health practice?**

Persistent disparities in vaccination coverage by health insurance status, race and ethnicity, and poverty status indicate that improvement is needed to achieve equity in the national childhood vaccination program. Efforts by health care providers and parents are needed to ensure that all children are protected from vaccine-preventable diseases.

## Footnotes

*
https://www.cdc.gov/vaccines/vac-gen/howvpd.htm

† Estimates for states, selected local areas, and the territories of Guam and Puerto Rico are available online at https://www.cdc.gov/vaccines/imz-managers/coverage/childvaxview/data-reports/index.html. Certain local areas that receive federal Section 317 immunization funds are sampled separately and included in the NIS-Child sample every year (Chicago, Illinois; New York City, New York; Philadelphia County, Pennsylvania; Bexar County, Texas; and Houston, Texas). Other local areas in Texas were sampled in some survey years from 2018–2020 and not others, including El Paso County (survey year 2019) and Dallas County (survey year 2019). Data were not collected in Puerto Rico in 2018 because of the severity of the hurricane season. Therefore, estimates for Puerto Rico are based on data collected in survey years 2019 and 2020. National estimates in this report exclude all territories.

§ The combined 7-vaccine series (4:3:1:3*:3:1:4) includes ≥4 doses of diphtheria, tetanus toxoids, and acellular pertussis vaccine; ≥3 doses of poliovirus vaccine; ≥1 dose of measles-containing vaccine; ≥3 or ≥4 doses (depending upon product type) of *Haemophilus influenzae* type b conjugate vaccine; ≥3 doses of hepatitis B vaccine; ≥1 dose of varicella vaccine; and ≥4 doses of pneumococcal conjugate vaccine.

¶ The NIS-Child used a landline-only sampling frame from 1995 through 2010. From 2011 through 2017, the survey was conducted using a dual-frame design, with both mobile and landline sampling frames included. In 2018, the NIS-Child returned to a single-frame design, with all interviews conducted by mobile telephone.

** Children with at least one vaccination reported by a provider and those who had received no vaccinations were considered to have adequate provider data. “No vaccinations” indicates that the vaccination status is known because the parent or guardian indicated there were no vaccinations, and the providers returned no immunization history forms or returned them indicating that no vaccinations had been given.

†† The Council of American Survey Research Organizations (CASRO) household response rate is calculated as the product of the resolution rate (percentage of the total telephone numbers called that were classified as nonworking, nonresidential, or residential), screening completion rate (percentage of known households that were successfully screened for the presence of age-eligible children), and the interview completion rate (percentage of households with one or more age-eligible children that completed the household survey). The CASRO household response rate is equivalent to the American Association for Public Opinion Research type 3 response rate (https://www.aapor.org/AAPOR_Main/media/publications/Standard-Definitions20169theditionfinal.pdf). CASRO response rates and the proportions of children with household interviews that had adequate provider data for survey years 2015–2019 are available at https://www.cdc.gov/vaccines/imz-managers/nis/downloads/NIS-PUF19-DUG.pdf.

§§ 45 C.F.R. part 46.102(l)(2); 21 C.F.R. part 56; 42 U.S.C. Sect. 241(d); 5 U.S.C. Sect. 552a; 44 U.S.C. Sect. 3501 et seq.

¶¶ MSA status was determined based on household reported city and county of residence and was grouped into three categories: MSA principal city, MSA nonprincipal city, and non-MSA. MSAs and principal cities were as defined by the U.S. Census Bureau (https://www.census.gov/programs-surveys/metro-micro.html). Non-MSA areas include urban populations not located within an MSA as well as completely rural areas.

***
https://www.cdc.gov/vaccines/imz-managers/coverage/childvaxview/pubs-presentations/NIS-child-vac-coverage-2018-2020-tables.html#figure-01

†††
https://www.cdc.gov/vaccines/imz-managers/coverage/childvaxview/pubs-presentations/NIS-child-vac-coverage-2018-2020-tables.html#figure-02

§§§ https://www.cdc.gov/vaccines/programs/vfc/. Eligible children include those aged ≤18 years who were Medicaid-eligible, uninsured, American Indian or Alaska Native, or insured by health plans that do not fully cover routine immunization.

¶¶¶
https://www.cdc.gov/vaccines/imz-managers/nis/downloads/NIS-PUF19-DUG.pdf

****
https://www.cdc.gov/vaccines/imz-managers/coverage/childvaxview/pubs-presentations/NIS-child-vac-coverage-2018-2020-tables.html#table-01
